# Assessment of Vertical Force Generated with Single File Systems during Shaping of Constricted Root Canals

**DOI:** 10.1055/s-0044-1786840

**Published:** 2024-07-16

**Authors:** Abdulmohsen Alfadley, Ahmed Jamleh

**Affiliations:** 1 Department of Restorative and Prosthetic Dental Sciences, College of Dentistry, King Saud bin Abdulaziz University for Health Sciences, King Abdullah International Medical Research Center, Ministry of National Guard Health Affairs, Riyadh, Kingdom of Saudi Arabia; 2 Department of Restorative Dentistry, College of Dental Medicine, University of Sharjah, Sharjah, United Arab Emirates

**Keywords:** HyFlex EDM file, OneShape, ProTaper NEXT, vertical force

## Abstract

**Objectives** This study aimed to evaluate the shaping force generated with OneShape (OS) and HyFlex EDM (HEDM) systems designed for single file shaping, in comparison with ProTaper Next (PTN).

**Materials and Methods** Maxillary premolar teeth received access cavity preparation and their canals were shaped with OS, HEDM, or PTN to size 25 according to manufacturer's instructions with consistent pressure on the files to give a gentle “in-and-out” movements of 2 mm amplitude. The canal shaping was completed with a total of three insertions. After each insertion, 1% NaOCl irrigation and recapitulation with K-file size 15 were performed. The vertical shaping force was measured using a force gauge (M5-20 Advanced Digital Force Gauge; Mark-10 Corporation, NY).

**Statistical Analysis** The shaping time was analyzed by using a one-way analysis of variance (ANOVA) test and differences between the mean apical and coronal maximum force values were analyzed using the Kruskal–Wallis and Mann–Whitney U tests. The level of significance was set as 0.05.

**Results** The magnitude of the vertical forces increased with successive advancements of the instruments within the canal. During canal shaping procedures in all groups, the apical and coronal maximum force values of the OS and HEDM ranged from 2.5 to 7.2 N and 1.3 to 2.9 N, respectively. PTN generated the lowest maximum apical forces during the second and third insertions ( *p* < 0.05). HEDM generated significantly less maximum coronal forces than both OS and PTN during the first insertion while the use of OS was associated with the highest amount of force values in the second and third insertions ( *p* < 0.05). In terms of shaping time, no significant differences were detected among the three tested systems ( *p* = 0.606).

**Conclusion** The tested single file systems were associated with higher shaping forces in the apical direction that were significant in the second and third insertions.

## Introduction

Superior chemomechanical debridement of the intricate root canal system is essential for the attainment of optimal endodontic treatment outcomes. 1 Canal shaping procedures aim to develop a continuously tapering root canal that facilitates the delivery of chemical irrigating solutions and intracanal medicaments as well as three-dimensional obturation of the root canal system. 2 Nowadays, engine-driven nickel–titanium (NiTi) files are predominantly used to shape root canals due to superior flexibility, improved cutting efficiency, proven canal centering ability, reduced instrumentation errors, superior cyclic and torsional fatigue resistance, reduced treatment time, and favorable treatment outcome compared with hand instrumentation with stainless steel files. 3 4 5 Despite such advantages, fracture of NiTi instruments may occur during clinical use as a result of either cyclic or torsional fatigue, or a combination of both mechanisms. 3 6 Such an incident can impede the cleaning and shaping of the apical portion of the root canal system which, in turn, may affect the outcome of the case. 7 Therefore, various changes have been incorporated into the manufacturing process of NiTi instruments to enhance the clinical performance and safety of root canal shaping procedures. Such enhancements include alterations in file design, thermomechanical treatment, motion kinematics, and electropolishing. 5 8

Single file NiTi systems have gained widespread acceptance among dental practitioners owing to reduced cost, instrumentation time, and cross-contamination risks. 9 The use of single file instruments was found to be equivalent to traditional multifile systems in terms of canal cleanliness, although it took less time to complete the shaping procedure. 10 11 OneShape system (OS; MicroMega, Besançon, France) is a single file NiTi rotary system (25/.06) that is made of conventional NiTi alloys. OS is characterized by having an asymmetric horizontal cross-sectional geometry with three sharp cutting edges and a nearly triangular cross-section in the tip region. Near the shaft, the instrument design changes into an S-shaped cross-section with two cutting edges. 10 HyFlex EDM file (HEDM; Coltene/Whaledent AG, Altstatten, Switzerland) is a new-generation single file instrument that works in continuous rotation. Like HyFlex CM files (Coltene/Whaledent AG), HEDM instruments are manufactured from a controlled memory alloy using electrodischarge machining technology. HEDM OneFile (25/∼) has a constant taper of 0.08 in the apical 4 mm of the file but that decreases to 0.04 in the coronal part of the instrument. This novel instrument has three different cross-sectional designs over the length of its working part: a quadratic cross-section near the tip, a trapezoidal cross-section in the middle area, and an almost triangular cross-section near the shaft. The hardened surface created by the EDM process and the variable cross-sectional design of the instrument increase the cutting efficiency and fatigue life of the instrument. 12

It is known that during root canal shaping, NiTi files are subjected to repeated cycles of multidirectional loadings which negatively affect the fatigue resistance of the instrument resulting in file fracture and other intraradicular defects such as root canal aberrations and dentinal cracks. 11 13 14 The shaping forces generated during root canal preparation can be influenced by different factors such as the contact area between the file and the canal walls, instrument design and geometry, preoperative canal size, file's motion kinematics, and forces exerted by the clinician. 5 15 16 17 18 19 Hence, assessing the magnitude of vertical forces generated during root canal shaping procedures is essential. The shaping force generated with single file systems during root canal preparation has not been adequately studied. Therefore, this study was conducted to assess the vertical forces produced with OS and HEDM single file systems and compare them with ProTaper Next (PTN) during canal shaping. The null hypothesis was there would be no difference between the three tested systems in the vertical forces generated during canal shaping.

## Materials and Methods

### Selection of Teeth and Preparation of Samples

This study was performed in accordance with the ethical guidelines of the Institutional Review Board (RC17/008/R) and the principles of Declaration of Helsinki. Before the commencement of the study, estimation of required sample size was established based on initial piloting. The sample size was determined using a 5% significance level and 80% power to detect a 2.1 N force difference between the three tested systems. The standard deviation was set at 2 N. According to this, 15 root canals were included for each group.

In this experiment, sound and fully formed two-rooted maxillary premolar teeth with two distinct canals starting at pulp chamber floor level were chosen from a pool of extracted human teeth that were maintained in distilled water. Facial and proximal digital periapical radiographs were obtained using Planmeca ProX™ (Planmeca®, Helsinki, Finland) to confirm the presence of mature roots, constricted canals, and relatively straight root canals (less than 10-degree curvature). 20

Access cavity preparation was accomplished and the canals were checked for patency and size. The working length (WL) was determined by placing a size 10 K-file into the canal until the tip of the instrument became flush with the apical foramen. The distance from the reference point in the occlusal surface to the file tip minus 0.5 mm was considered the WL of the canal. The root canal was considered sufficiently constricted by placing a size 15 stainless steel file that would bind 3 mm or more away from the canal length. If these criteria were not met, the tooth was excluded from the study sample. Hence, 45 root canals were selected for this study. Thereafter, the apical root tip was covered with utility wax, and the whole tooth was embedded in a mixed autopolymerizing resin (DuraLay; Reliance Dental Manufacturing Co, Worth, IL) that enclosed the root surface along with the surrounding wax and left to set in room temperature. After the complete resin setting, a manual glide path was established for each canal using sizes 10 and 15 K-file. Throughout the glide path preparation phase, canals were irrigated with 1% NaOCl followed by a final rinse with saline. Afterward, each sample was wrapped in moist gauze and stored in a test tube.

### Vertical Force Measurements

**Fig. 1:**
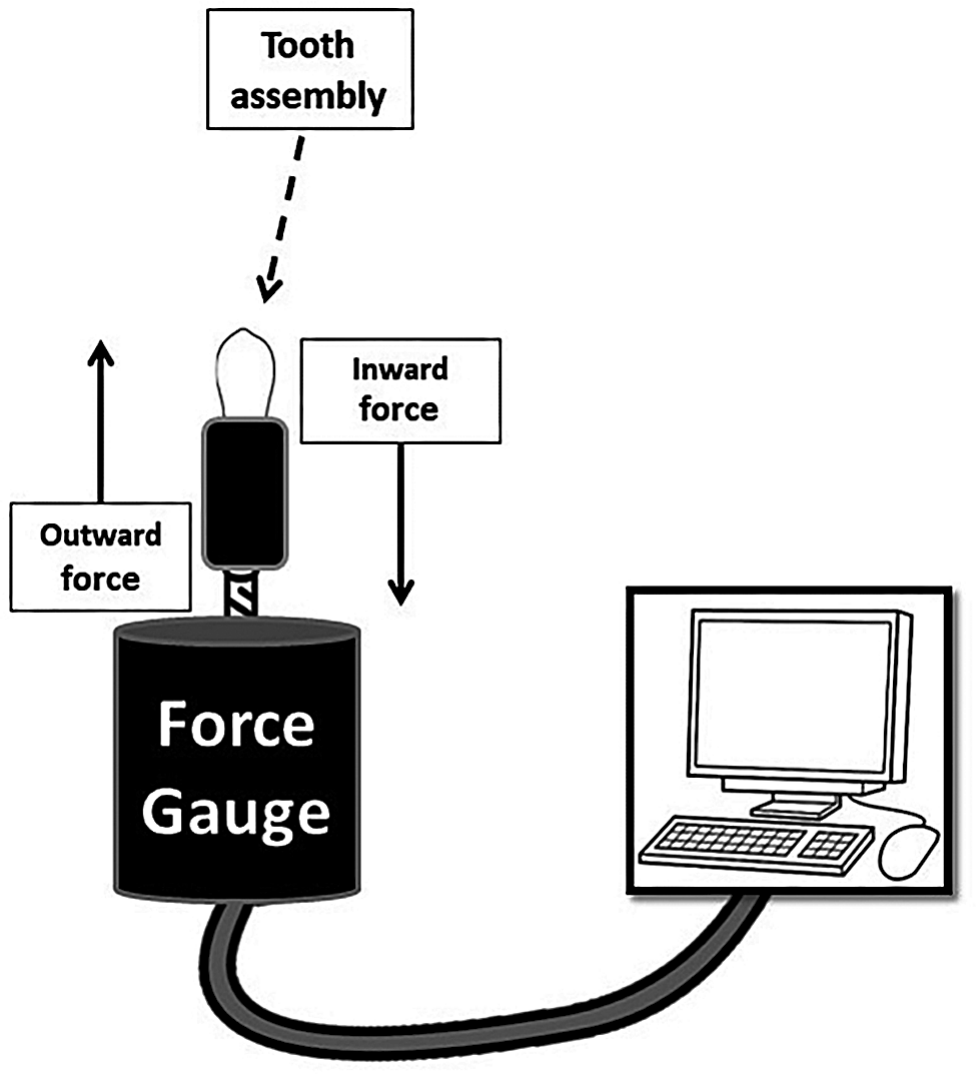
Schematic drawing of the experimental setup. The force gauge was used for recording vertical forces in inward and outward directions and the received data are presented on a personal computer using MESUR Lite software (Mark-10 Corporation).

A force gauge (M5-20 Advanced Digital Force Gauge; Mark-10 Corporation, Long Island, NY) with a force capacity of 100 N and a capability to measure data every 0.1 second was used to assess canal shaping forces in real time ( Fig. 1 ). This force gauge is designed for tension and compression testing across various industries. The device features a sampling rate of 7,000 Hz making it capable to produce accurate readings even for quick-action test. Accuracy is ±0.1% of full scale while resolution is 1/5,000.

The generated forces during canal shaping procedures were displayed using the MESUR™ Lite software (Mark-10 Corporation, NY). Before the commencement of canal shaping for each sample, each tooth was set on a fixed stage and the force gauge was zeroed. The vertical shaping forces generated during canal shaping were obtained in both apical (inward) and coronal (outward) directions. The vertical force data were expressed in newtons.

### Root Canal Shaping

Following the preparation of a manual glide path to WL with a size 15 K-file, the root canals were randomly assigned based on their WL to three similar experimental groups; OS, HEDM, and PTN systems ( *n* = 15 each). The average WLs of the OS, HEDM, and PTN groups were 20.57 ± 1.43, 21.20 ± 1.81, and 21.07 ± 2.05 mm, respectively. There were no statistically significant differences in WLs among the three groups ( *p* = 0.594).

All canal shaping procedures were performed using the Element Motor (SybronEndo, Orange, CA) under continuous rotary motion. Canals in the OS group were shaped with the file size 25/.06 at a speed of 400 rpm. In the HEDM group, the OneFile instrument was used to complete canal shaping at a speed of 500 rpm. In the two groups, each canal was shaped with one file inserted three times until reaching the WL. Each insertion cycle incorporated a maximum of three pecking motions. In the PTN group, canal shaping was completed using an X1 file inserted once into WL, followed by an X2 file inserted into WL through two insertions at a speed of 300 rpm. In agreement with the manufacturer's recommendations, the torque setting was standardized for all experimental groups at a value of 2.5 Ncm. Shaping procedures were completed using gentle in-and-out movements under copious irrigation with 1% NaOCl.

After each instrument insertion, the canal was irrigated with 1 mL of 1% NaOCl, apical patency was verified using a size 10 K-file, and the file's flutes were cleaned with alcohol swab. Each rotary file was used to shape a maximum of four root canals unless evidence of deformation or fracture was noted earlier. The root canals were shaped by a single experienced operator. The mean force values of the three shaping insertions were used for comparison. The shaping time, defined as the active instrumentation time, was recorded.

### Data Analysis

Since the vertical force data were not normally distributed (Shapiro–Wilk test; *p* < 0.05), differences between the mean coronal and apical maximum force values were analyzed using the Kruskal–Wallis and Mann–Whitney U tests.

The WL of selected teeth and canal shaping time were tested for statistical significance using a one-way ANOVA test. All statistical analyses were performed using SPSS software version 22 (SPSS Inc, Chicago, IL). The level of significance (α) was set as 0.05.

## Results

**Fig. 2:**
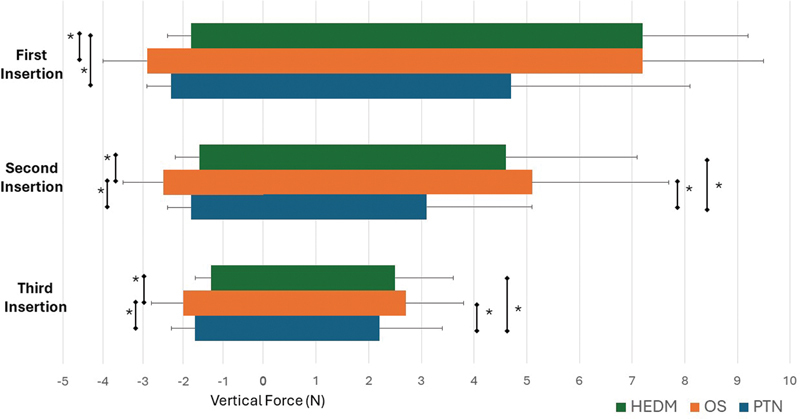
The means and standard deviations of apical (positive values) and coronal (negative values) forces for all insertions in the tested systems. The asterisk (*) indicates statistical significance. HEDM, HyFlex EDM; OS, OneShape; PTN, ProTaper Next.

In this study, each canal was shaped through three file insertions. The magnitude of the vertical forces increased with successive advancements of the files within the canal ( Fig. 2 ). During canal shaping procedures in all groups, the apical and coronal maximum force values ranged from 2.2 to 7.2 N and 1.3 to 2.9 N, respectively. There were significant differences in maximum force values among the tested groups at each insertion.

For forces generated during apical file advancement, PTN generated the lowest peak loads during the second and third insertions ( *p* < 0.05). For vertical forces induced during coronal withdrawal of the instrument, HEDM generated significantly less maximum forces than both OS and PTN during the first insertion while the use of OS was associated with the highest amount of force values in the second and third insertions ( *p* < 0.05).

The average shaping times of the OS, HEDM, and PTN groups were 21.95, 23.61, and 21.91 seconds, respectively, with no significant differences between the groups ( *p* = 0.606). No file fracture or plastic deformation was encountered throughout the experiment.

## Discussion

Safe and effective chemomechanical preparation of the root canals relies on the endodontic file performance. 21 Single file systems produce predictable canal cleanliness with reduced instrumentation time and cost. 11 22 23 Based on this, the current study was conducted to provide data on the vertical forces generated with OS and HEDM during the shaping of narrow canals, in comparison with PTN.

OS and HEDM systems successfully shaped the root canals with only one file with no prior shaping with smaller files. This results in a large contact surface with the canal walls that would generate great stresses and strains on the dentin and file. 24 This is evident in the current findings wherein the magnitude of the vertical forces increased with successive use of the files within the canal. Consistently, previous studies showed that more forces are needed to reach the WL in single file systems or to shape the canal with a larger file size in multifile systems. 15 16 24 25 26

It should be mentioned that this study results should be interpreted with caution. Considering that the roots were fixed to acrylic resin and the forces were transmitted to the gauge through the acrylic resin, the observed values are probably greater than the forces generated in a clinical setting. In a clinical scenario, the periodontal ligament and surrounding bone would disperse the forces. Nonetheless, the testing conditions were standardized throughout the experiment and differences in vertical forces were shown between the tested systems in shaping narrow canals. In terms of the apical forces, OS and HEDM generated apical forces that exceeded 7 N in the last insertion. The results were found to be higher than PTN in the second and third insertions. Similarly, a previous study showed that apical forces generated with WaveOne and WaveOne Gold single file systems were high. 17 Another study showed that multifile systems generated apical forces of no more than 6.4 N. 26 This supports the idea that shaping the canal successively with a smaller file size led to lower forces generated by the last file and that canal shaping with single file systems has been mostly based on simplicity but not proven effectiveness. 11 Nevertheless, a recent study revealed that preshaping canal with a smaller file could reduce the generated forces induced during the shaping under reciprocating motion. 27

The “screwing-in” effect of the examined files was tested by measuring how much force was needed to pull the file coronally. 28 It may cause file penetration beyond the apex, leading to overshaping and cracks on the apical root surface. 29 The results showed significant differences between OS and HEDM in the three insertions. Although the HEDM file had a larger taper in the apical part, its rotational speed is lower than the OS file. It has been reported that the rotational speed affects the contact time of the file with the canal walls. 30 The high speed releases the close binding of cutting edges to the canal wall which reduces the screwing-in force. 30 This could explain the favorably low coronal forces required to shape the canal with HEDM.

Clinically, the shaping procedure creates unavoidable stresses mainly at or near the file tip. 31 This stress induces strain on the canal walls that would lead to the development of dentinal defects. 24 29 32 Moreover, it has been shown that the shaping force generates operational torque that creates higher stresses on the file, resulting in file fracture. 6 13 25 Therefore, gentle shaping is needed. 33 Although higher vertical forces were observed with the tested single file systems (OS and HEDM) compared with PTN, no plastic deformation or fracture took place. This result may indicate that the OS and HEDM are resistant to fracture and can shape up to four narrow canals using the described shaping technique. This is in agreement with previous findings. 17 23

In this study, there are certain limitations that must be acknowledged. First, the study investigated only one aspect of file behavior, which is the vertical force induced during canal shaping procedure. Hence, future experiments should examine the effect of generated force on other attributes such as associated torque, file deformation, and fracture, as well as on the root canal wall. Second, despite the variations in root canal morphology of natural teeth, the research team attempted to maintain comparability of the experimental groups. For instance, only defect-free two-rooted maxillary premolar teeth with two constricted and relatively straight canals starting at pulp chamber floor level were considered for inclusion in the study, and the three groups were balanced with respect to the WL. Another limitation of this study relates to the possible differences in pressure exerted by the operator during canal shaping. Future studies should consider using automated devices to allow for consistent vertical pressure and pecking motion to eliminate the potential for operator bias. Nevertheless, canal shaping was performed by one experienced operator who maintained gentle “in-and-out” movements of 2 to 3 mm amplitude each to ensure consistency across the experimental groups.

## Conclusion

Within the current study parameters, the amount of vertical forces increased with the progressive advancements of the instruments within the canal. PTN generated the lowest maximum apical forces during the second and third insertions. HEDM generated significantly less maximum coronal forces than both OS and PTN during the first insertion while the use of OS was associated with the highest amount of force values in the second and third insertions. Thus, the null hypothesis was rejected.

Further investigations should be conducted to simultaneously examine the effect of developed vertical forces on instrument performance such as associated torque and fatigue behavior as well as on the canal structure in other groups of teeth with wider variety of pulp space features.
